# Efficacy of recombinant human interleukin-11 in preventing and treating oral mucositis after chemotherapy for patients with acute leukemia

**DOI:** 10.1186/s12903-023-03118-4

**Published:** 2023-07-12

**Authors:** Yilin Zhang, Yuxin Li, Aili He, Jin Wang, Pengyu Zhang, Bo Lei, Zhuan Huang, Lin Zhang, Wei Zhao, Xiaorong Ma

**Affiliations:** Department of Hematology, The Second Affiliated Hospital of Xi’an Jiaotong University, Xi’an, Shaanxi 710004 China

**Keywords:** Acute leukemia, Oral mucositis, Recombinant human interleukin-11

## Abstract

**Objective:**

This study aimed to investigate the clinical effects of recombinant human interleukin-11 (rhIL-11) gargle on preventing and treating oral mucositis (OM) after chemotherapy for acute leukemia.

**Methods:**

This single-site, prospective, observer-blinded, nonrandomized controlled trial was conducted on 74 patients with acute leukemia, who were divided into the experimental and control groups. The patients in the experimental group were treated with IL-11 gargle, and those in the control group were treated with sodium bicarbonate gargle. We examined the time and severity of oral mucositis, severity and duration of associated pain, healing time of mucositis, effects of OM on eating, and levels of T-cell subset indicators before and after treatment to evaluate the effects of IL-11 treatment.

**Results:**

The proportion of patients with severe OM was significantly lower in the experimental group than in the control group. Mucositis occurred later in the experimental group compared with the control group. The degree and duration of pain, ulcer healing time, and effects on eating were lower in the experimental group compared with the control group. Following treatment, the levels of all T-cell subset indicators improved in each of the two groups. However, the rate of improvement was significantly higher in the experimental group than in the control group. These differences were statistically significant (*P* < 0.05).

**Conclusions:**

IL-11 gargle reduced the severity of OM after chemotherapy for acute leukemia. Treatment with IL-11 relieved pain, promoted healing, and improved the curative effect of the condition, making it worthy of clinical promotion.

## Introduction

Oral mucositis (OM) is an inflammatory reaction of epithelial tissue that involves symptoms such as oral mucosal erythema and ulcers. The main antitumor treatments that cause OM include chemotherapy, radiotherapy, molecular targeted therapy, and hematopoietic stem cell transplantation for malignant tumors [1–4]. OM caused by chemotherapy, also known as chemotherapy-induced OM (CTOM), usually occurs 4–7 days after the start of chemotherapy, peaking between days 10 and 14 [5]. The main manifestations of CTOM include oral mucosal congestion, erythema, edema, different degrees of erosion and ulcers, local pain, difficulty eating, dry mouth, and dysgeusia. These symptoms severely impair the quality of life and antitumor efficacy among patients with CTOM [6–8].

Acute leukemia is a cancer of the hematopoietic system. At present, chemotherapy remains the primary treatment method for the disease. However, acute leukemia chemotherapy and the drugs used for treatment can result in bone marrow suppression, low immunity, and systemic infection in severe cases. In turn, this has caused OM to become the main nonhematological complication of leukemia [9, 10]. Therefore, active prevention and treatment of the condition are crucial. Recombinant human interleukin-11 (IL-11) is a pleiotropic cytokine that promotes myeloid hematopoiesis, inhibits immune activation, stabilizes the internal environment, and protects the mucosal epithelium. This pluripotent synergy can significantly improve chemotherapy-induced oral mucosal inflammation [11, 12]. The main purpose of this study was to investigate the preventive and therapeutic effects of IL-11 oral gargle on OM in patients with acute leukemia following chemotherapy.

## Patients and methods

### Patients

#### Patient demographics

This study involved 74 patients with acute leukemia (including secondary and transformed) admitted to our hospital from June 2021 to June 2022. This group consisted of 59 patients with acute myeloid leukemia (AML), who all met the diagnostic criteria for adult AML from the Chinese Medical Association [13], and 15 patients with acute lymphoblastic leukemia (ALL), who all met the diagnostic criteria for adult ALL from the Chinese Medical Association [14]. The 74 patients were nonrandomly divided between the experimental and control groups, with 37 patients in each group. All the patients were observed for 23–35 days.

#### Inclusion criteria

Patients were included in this study if they (1) had AML or ALL diagnosed by bone marrow aspiration and flow immunophenotyping and receiving the first course of induction chemotherapy; (2) were diagnosed with acute leukemia, after complete response (CR), and receiving a course of consolidation and intensive chemotherapy; and (3) could take care of themselves, with good dental and periodontal cleanliness, and had good treatment compliance. During treatment, they followed daily oral care and were guided by dental doctors.

#### Exclusion criteria

Patients were excluded from this study if they (1) had active periodontal diseases that required treatment and refractory oral ulcers prior to this study; (2) were pregnant and lactating; (3) presented with other malignant tumors; (4) had other infectious diseases; or (5) had severe heart, brain, lung, liver, kidney, or other diseases that prevented them from tolerating chemotherapy.

### Methods

#### Chemotherapy regimens

Each patient in this study was treated with a reasonable chemotherapy regimen based on their own conditions. Drugs commonly used to treat AML include idarubicin, cytarabine, high-spinel, etoposide, fludarabine, cladribine, azacitidine, decitabine, arsenic acid, venetoclax, and other novel chemotherapy drugs. Drugs commonly used to treat ALL include cyclophosphamide, vincristine, pirarubicin, methotrexate, cytarabine, dexamethasone, pegaspargase, etoposide, tyrosine kinase inhibitors imatinib, dasatinib, and other novel chemotherapy drugs. The choice of chemotherapy drugs was standardized for patients within each group, implying that these patients were not treated significantly differently.

#### OM treatment

As part of the intervention, the patients in the experimental group gargled with IL-11 rinse, which was formulated as follows: 3 mg of IL-11 was added to 100 mL of normal saline, which was then stored at 2–8 °C and dispensed every day. The patients in the control group gargled with a solution of 5% sodium bicarbonate and purified water at a ratio of 1:1. The patients in both groups gargled with their respective solution in the morning, after lunch, after dinner, and half an hour before going to bed for 4 days. Each time, they rinsed with water before gargling and gargled with about 25 mL of solution for 5 min. The intervention began on day 1 of chemotherapy and ended after bone marrow suppression phase remission (detachment of granulocyte deficiency) or after their oral mucosal ulcers had healed.

#### Observation indicators

The main observation indicators used in this study were as follows: (1) the severity of OM and its timing relative to the first day of chemotherapy; (2) the severity of the pain caused by OM and the duration from the onset of pain to its relief; (3) the time from the inception of OM to its healing; (4) the influence of OM on eating, where patients with reduced food consumption, liquid food intake, or an inability to eat due to OM erosion or pain were considered to have an impact on eating and the rest were not; and (5) T-cell subsets of patients detected at the start of agranulocytosis or OM. As with the other indicators examined before treatment, T-cell subsets were re-examined after oral gargle treatment, and the differences between the two groups were compared.

According to the World Health Organization (WHO) [1] classification, OM is classified as follows: Grade 0, normal oral mucosa; Grade I, one or two ulcers less than 1 cm in diameter, mild pain symptoms, and no influence on eating; Grade II, one large ulcer (> 1 cm) and several small ulcers, worsened pain symptoms, and limited impacts on eating; Grade III, two large ulcers (> 1 cm) and several small ulcers, which could only be fed; and Grade IV, more than two large ulcers (> 1 cm) fused into pieces and the patient unable to eat. The degree of pain was assessed using the visual analogue scale, which was defined as follows: painless, 0; mild pain, 1–3; moderate pain, 4–6; and severe pain, 7–10.

Physicians with experience in the treatment of oral diseases evaluated the severity of OM and degree of pain. Unaware of the disease groupings, the same physician assessed the entire observational study process.

### Statistical analysis

SPSS 20.0 software was used to perform all statistical analyses. The continuous data were expressed as mean ± standard deviation (x ± s) and compared using the *t* test. The count data were expressed as frequency and compared using the *χ*^2^ test. A *P* value < 0.05 indicated a statistically significant difference.

## Results

### Patient characteristics

**Fig. 1:**
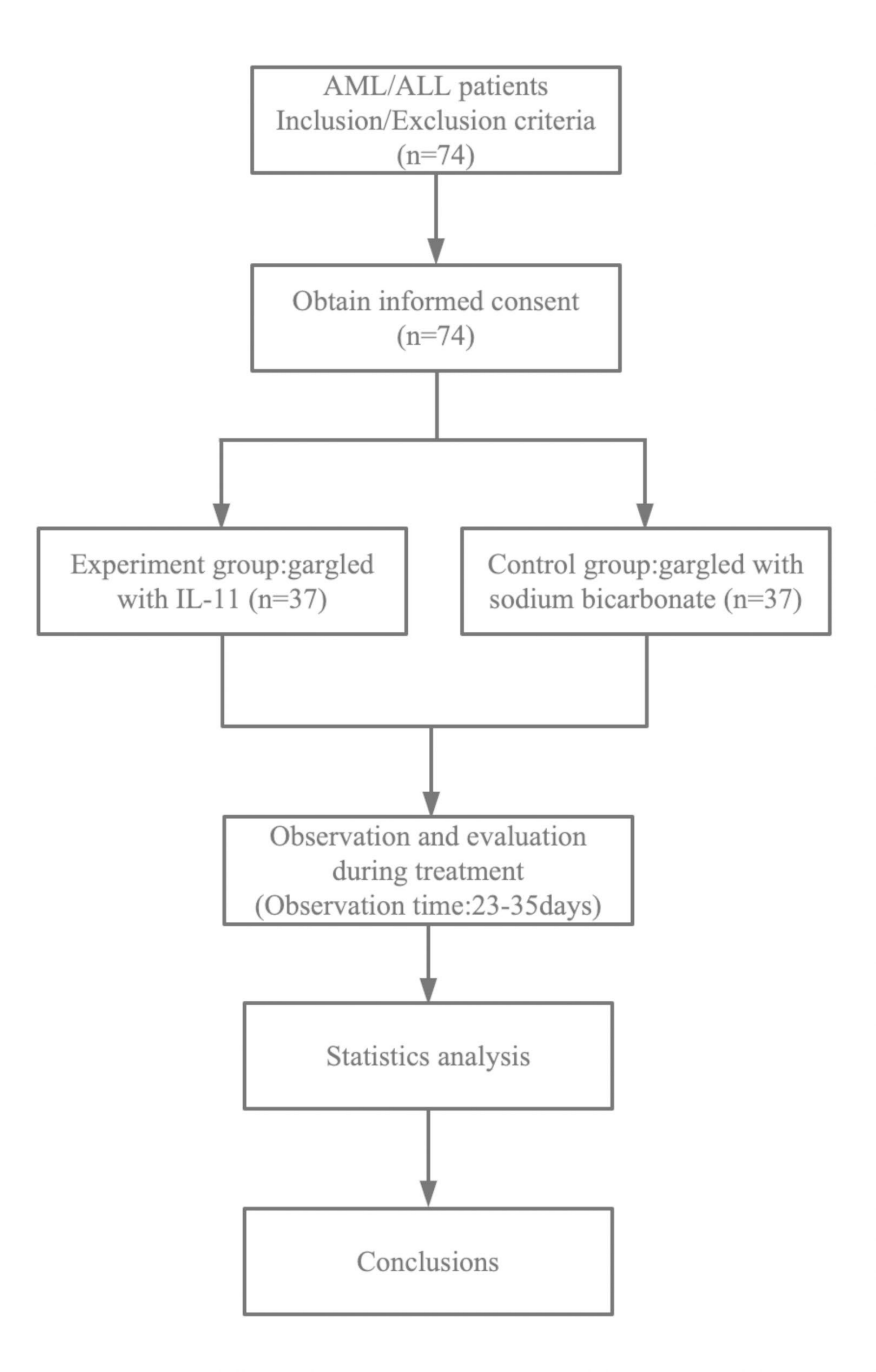
Patients inclusion, exclusion, and follow-up

The 74 patients were divided between the experimental and control groups, with 37 patients in each group. Patient inclusion, exclusion, and follow-up are shown in Fig. 1. Their baseline clinical data are shown in Table 1. The control group comprised 20 male and 17 female patients, aged 18–74 years, with a median age of (48.2 ± 9.3) years. The study included 30 patients with AML, 7 patients with ALL, 14 cases of initial diagnosis, 23 cases after CR, and 5 patients with dentures. The experimental group comprised 18 male and 19 female patients, aged 17–76 years, with a median age of (48.9 ± 8.9) years. The study comprised 29 patients with AML, 8 patients with ALL, 14 cases of initial diagnosis, 23 cases after CR, and 6 patients with dentures. We did not observe any significant differences between the two groups in terms of baseline clinical data, such as age, sex, type, disease state, and use of dentures (*P* > 0.05). All patients provided written informed consent prior to treatment.

**Table 1:** Comparison of baseline clinical data between the observation and control groups

Group		Control	Experimental	*P* value
*n*		37	37	
Age		48.2 ± 9.3	48.9 ± 8.9	>0.05
Gender	Male	20	18	>0.05
	Female	17	19	
Diagnosis	AML	30	29	>0.05
	ALL	7	8	
Disease state	Initial diagnosis	14	12	>0.05
	Complete response	23	25	
Use of dentures	Yes	5	6	>0.05
	No	32	31	

### Severity and timing of OM

More patients in the experimental group had OM of grade 0 and grade I, whereas more patients in the control group had OM of grade II and above (*P* < 0.05). Additionally, OM occurred later in the experimental group compared with the control group (*P* < 0.05) (Table 2).

**Table 2:** OM stage and timing after chemotherapy

Group	*n*	Stage of OM response					OM occurrence time (days)
		0	I	II	III	IV	
Control	37	8	10	12	5	2	6.38 ± 2.45
Experimental	37	14	15	5	2	1	8.63 ± 2.14
*P* value		< 0.05					< 0.05

### Level and duration of OM pain

The patients in the experimental group mostly experienced painlessness or mild symptoms of pain, whereas the patients in the control group had significantly higher levels of moderate and severe pain (*P* < 0.05). Additionally, IL-11 treatment significantly shortened the duration of pain (*P* < 0.05) (Table 3).

**Table 3:** Level and duration of OM pain

Group	n	Pain level				Pain duration (days)
		Painless	Mild	Moderate	Severe	
Control	37	7	10	14	6	9.03 ± 3.12
Experimental	37	13	16	7	1	6.58 ± 2.46
*P* value		<0.05				<0.05

### Effects of OM on eating and mucosal healing time

The onset of OM had a significantly higher impact on eating in the control group compared with the experimental group (*P* < 0.05). Furthermore, the healing time was much shorter for the experimental group compared with the control group (*P* < 0.05) (Table 4).

**Table 4:** OM effects on eating and healing time

Group		n	Eating effects		Healing time (days)
			Influential	No Impact	
Control		37	22	15	11.63 ± 4.21
Experimental		37	13	24	7.89 ± 3.84
*P* value			<0.05		<0.05

### T-cell subset detection before and after treatment

Prior to treatment, the CD3+, CD4+, CD8+, and CD4+/CD8 + levels did not significantly differ between the two groups. The levels of each of these indicators increased as a result of IL-11 treatment, where the rate of increase in the experimental group was significantly higher than that in the control group (*P* < 0.05) (Table 5).

**Table 5:** T-cell subset detection before and after treatment (%)

Group	*n*	CD3 (+)		CD4 (+)		CD8 (+)		CD4 (+)/CD8(+)	
		Pre-treatment	Post-treatment	Pre-treatment	Post-treatment	Pre-treatment	Post-treatment	Pre-treatment	Post-treatment
Control	37	65.48 ± 5.45	78.63 ± 4.19	32.98 ± 3.46	46.45 ± 6.56	28.51 ± 4.33	38.70 ± 5.57	0.97 ± 0.35	1.87 ± 0.45
Experimental	37	64.53 ± 5.67	84.25 ± 3.95	31.77 ± 4.10	42.18 ± 7.12	29.15 ± 3.97	34.47 ± 6.24	1.02 ± 0.28	1.65 ± 0.39
*P* value		>0.05	<0.05	>0.05	<0.05	>0.05	<0.05	>0.05	<0.05

## Discussion

Acute leukemia is a malignant clonal disease involving hematopoietic stem cells. Abnormal primitive cells proliferate and extensively infiltrate the bone marrow to inhibit normal hematopoiesis. At present, combined chemotherapy remains the primary form of treatment for acute leukemia. Acute leukemia commonly causes OM due to the characteristics of the disease itself and the influence of chemotherapy drugs [15]. Acute leukemia infiltrates the hematopoietic system, leading to severe bone marrow suppression after chemotherapy. All patients in this study experienced agranulocytosis after chemotherapy, resulting in low immunity. Therefore, these patients were more susceptible to oral and systemic infections. Additionally, acute leukemia can easily infiltrate the gums and cause gingival swelling. This may cause bleeding in the gums and oral mucosa or even result in the formation of hematomas due to various reasons, including low platelet levels. Further infiltration and bleeding only aggravate OM. After chemotherapy, patients with leukemia often experience nausea, vomiting, poor appetite, decreased saliva secretion, and decreased oral self-cleaning ability. In turn, these symptoms can induce OM. Chemotherapeutic drugs commonly used for acute leukemia include methotrexate, idarubicin, cytarabine, cyclophosphamide, vincristine, and new chemotherapy and targeted drugs, which have also been found to cause post-chemotherapy OM [5, 16, 17]. Finally, patients with acute leukemia often use antibiotics for a long time due to prolonged agranulocytosis complicated by infection. This may result in dysbacteriosis, which further increases the incidence of OM. In this study, the incidence rate of grade I and above OM after chemotherapy for acute leukemia was 78.4% in the control group and 63.5%in the treatment group. This background and our results collectively emphasized the importance of the effective prevention and treatment of OM.

OM causes local redness, swelling and pain, difficulty chewing, and issues with the patient’s normal eating. If left untreated for a long time, these symptoms can lead to malnutrition, further weakening the patient’s resistance, exacerbating infections, intensifying pain, prolonging treatment time, and imposing a greater financial burden on the patient [18, 19]. Furthermore, OM may reduce the patient’s tolerance and compliance with antitumor treatment due to increased pain, forcing clinicians to prolong the chemotherapy cycle and reduce the intensity of chemotherapy. These factors significantly impact the disease remission rate and depth of remission among patients with acute leukemia, thereby affecting survival time.

IL-11 is an anti-inflammatory factor in the interleukin family. IL-11 not only stimulates the differentiation and maturation of hematopoietic progenitor cells (megakaryocytes, granulocytes, and erythroid cells) but also counteracts and regulates the inflammatory cytokines produced by infection and damage caused by inflammation through a negative feedback regulation mechanism. At the same time, IL-11 acts on epithelial cells, maintains metabolism and homeostasis, and protects and strengthens the mucosal epithelium, thereby reducing edema and inhibiting inflammatory responses [11, 12, 20]. As early as 2007, foreign scholars concluded that the application of IL-11 significantly reduced the incidence of infection in patients with AML after chemotherapy [21]. In 2018, a clinical study investigated the effects of IL-11 on patients during radiotherapy [22]. Oral mucosal damage and pain significantly reduced following IL-11 treatment in patients with OM. Chinese researchers examined the effects of IL-11 gargle and aerosol therapy on OM caused by leukemia chemotherapy and allogeneic hematopoietic stem cell transplantation [23, 24]. Their results indicated that the topical application of IL-11 significantly relieved the pain caused by OM, reduced the degree of OM development, and shortened the healing time for the condition.

In this study, patients with acute leukemia who were treated with chemotherapy were selected as research subjects to observe the occurrence time, severity, pain, degree, duration, mucosal healing time, and effects of OM after topical treatment with IL-11 gargle on eating. Our results showed that IL-11 treatment could not completely prevent the occurrence of OM, but did reduce the incidence and grade of the condition. Furthermore, IL-11 treatment lowered pain caused by OM, reduced its impact on eating, and promoted mucosal recovery. Preventing OM complications not only relieved patients of potential pain but also ensured the intensity and efficacy of chemotherapy, thus improving the prognosis of acute leukemia. Overall, we concluded that IL-11 was effective in treating OM. However, further research is needed on the mechanism of IL-11 in treating mucositis. More specifically, future research should use subgroup analysis to better understand how IL-11 reduces the effects of cytokines and promotes the repair of mucosal epithelial cells.

Periodontal diseases such as pulpitis, periapical periodontitis, and periodontitis that already exist in patients before chemotherapy can also impact the occurrence and development of oral mucosal inflammation. We excluded patients with active periodontal diseases that required treatment before the study and tried to exclude the impact of this factor on the results. In this study, 11 elderly patients used dentures, including 6 in the IL-11 group and 5 in the control group. The friction and movement of dentures might cause oral mucosal inflammation and worsen oral mucosal inflammation, but no difference was found between the two groups and the analysis of later results was not affected.

Cell subsets, including CD3+, CD4+, and CD8 + cells, are important indicators for evaluating the immune function of the body. All T cells are positive for CD3 and further divided into CD4+, CD8+, and regulatory T cells based on their respective function. CD4 + cells mediate cellular immune responses and assist humoral immune responses. The main function of CD8 + cells is to kill target cells specifically and directly. CD4 + and CD8 + cells are in a constant state of balance and play an essential role in maintaining normal immune function. A decrease in the ratio of CD4 + to CD8 + cells indicates that the immune function of the body is in an imbalanced state, which may easily lead to various diseases including OM [25]. In this study, all indicators of T-cell subsets improved after treatment in both groups, where the rate of improvement was significantly higher in the experimental group compared with the control group. Previous studies showed that T-cell subsets might be directly involved in the formation of oral ulcers, showing different changes during different stages of oral ulcer development [26, 27]. Effective therapeutic drugs for OM promote the healing of oral mucosal ulcers and significantly improve the level of T-lymphocyte subsets. In this study, IL-11 gargle treatment effectively promoted the recovery of OM and significantly increased the levels of all T-cell subsets. Considering the potential effect of OM itself on T-cell subsets, future studies should further investigate whether the application of IL-11 alone increases the level of T-cell subset. Additionally, these investigations should aim to elucidate the underlying mechanism by which IL-11 operates in this context.

OM treatment excludes the application of anti-infection and biological factors, and includes other drug and nondrug therapies. For example, most of the patients with ALL had OM of grade III or above during the high-dose methotrexate course in this trial. The treatment for these patients included not only IL-11 but also local and systemic administration of leucovorin to reduce the damage caused by chemotherapy and speed up mucosal healing. In addition, various oral mucosa protective agents, oral care solutions, traditional Chinese medicine preparations, honey adjuvants, and other natural medicines can be used to improve the discomfort caused by OM, effectively reducing the incidence of mucositis and improving its remission rate [28–30]. Nonpharmaceutical treatment measures for OM are also extremely important because OM intervention should involve psychological, hygienic, nutritional, and habitual approaches. This may include improving oral hygiene after meals; strengthening general oral health; increasing the frequency of gargling with water to keep the mouth moist; eating less hard, spicy, or stimulating foods; eating more and less residue, moist food; and receiving psychological counseling.

The findings indicate that the incidence and severity of OM are affected by different chemotherapy or radiotherapy protocols [31]. Therefore, a wide heterogeneity is evident among oral health prevention programs dedicated to patients with cancer. Anti-inflammatory medications, growth factors and cytokines, cryotherapy [32], and laser-and-light therapy showed some degree of efficacy in preventing/reducing the severity of mucositis with most anticancer treatments. However, few studies explored the role of IL-11 in preventing and treating OM. Recombinant human IL-11 is a pleiotropic cytokine that promotes myeloid hematopoiesis, inhibits immune activation, stabilizes the internal environment, and protects the mucosal epithelium. This pluripotent synergy can significantly improve chemotherapy-induced oral mucosal inflammation, which is highly explorative [11, 12]. In addition, most current research is based on the study of OM after radiotherapy and chemotherapy for head and neck tumors; few studies have been performed on OM after chemotherapy for patients with acute leukemia [33]. Importantly, acute leukemia due to its own characteristics and the influence of chemotherapy drugs, resulting in its easy to combine OM. This study mainly explored the efficacy of recombinant human IL-11 in preventing and treating OM in patients with acute leukemia, with good therapeutic effects. Preventing OM complications not only relieves patients of potential pain but also ensures the intensity and efficacy of chemotherapy, thus improving the prognosis of acute leukemia.

This study had several limitations. First, it was a single-site, prospective, observer-blinded, nonrandomized controlled trial. It had small sample size and included patients who received treatment in our hospital, leading to bias in the analysis results. We need to further expand the sample size in future studies to increase the credibility of the findings. Second, the single application of IL-11 in treating oral mucosal inflammation had limited efficacy, and the combined effects of other drugs and nondrug treatments were not addressed in this study. Finally, the mechanism of IL-11 in treating oral mucosal inflammation needs further basic experimental research.

In summary, a precise prevention plan should be formulated according to risk stratification for treating OM after chemotherapy for acute leukemia. Besides IL-11 gargle, individualized prevention and treatment regimens for OM should incorporate other drugs and nonpharmaceutical measures. We may better relieve suffering and improve quality of life by providing personalized treatment plans according to the characteristics of the patient.

## Data Availability

All data generated or analysed during this study are included in this published article.

## Abbreviations

rhIL-11: Recombinant human interleukin-11
OM: Oral mucositis
CTOM: Chemotherapy-induced oral mucositis
AML: Acute myeloid leukemia
ALL: Acute lymphoblastic leukemia
WHO: World Health Organization
VAS: Visual analogue scale
